# The synergetic potential of *Lactobacillus delbrueckii* and *Lactobacillus fermentum* probiotics in alleviating the outcome of acute toxoplasmosis in mice

**DOI:** 10.1007/s00436-023-07787-6

**Published:** 2023-02-14

**Authors:** Tasneem M. Almallah, Safaa I. Khedr, Kholoud A. El Nouby, Salwa S. Younis, Mona A. Elazeem, Dalia A. Elmehy

**Affiliations:** 1 Medical Parasitology Department, Faculty of Medicine, Tanta University, Tanta, Egypt; 2 Medical Parasitology Department, Faculty of Medicine, Alexandria University, Alexandria, Egypt; 3 Pathology Department, Faculty of Medicine, Tanta University, Tanta, Egypt

**Keywords:** *Toxoplasma gondii*, Trimethoprim-sulfamethoxazole, Probiotics, *Lactobacillus*, Scanning electron microscopy, IFN-γ

## Abstract

Toxoplasmosis is an immunologically complex disease, particularly in immunocompromised patients. Although there are several therapeutic regimens for such disease, the majority of them have many drawbacks. Therefore, it is of utmost importance to improve the current regimen in an effort to achieve a well-tolerated therapy while also enhancing the host immune response. Famous for their immunomodulatory effect, *Lactobacillus delbrueckii* and *Lactobacillus fermentum* probiotics were chosen to be evaluated in this study as an adjuvant therapy against the virulent RH *Toxoplasma gondii* (*T. gondii*) strain. Experimental mice were divided into control and treated groups. The control group was further subdivided into two groups: group I: 10 uninfected mice and group II: 20 infected untreated mice. The treated experimental group was subdivided into three groups (20 mice each); group III: sulfamethoxazole-trimethoprim (SMZ-TMP) treated, group IV: probiotics treated, and group V: SMZ-TMP combined with probiotics. The results obtained revealed that combined therapy increased survival rate and time up to 95% and 16 days, respectively, with an 82% reduction of tachyzoites and marked distortion, as detected by the scanning electron microscope (SEM). Additionally, combined therapy alleviated the severity and the extent of the inflammatory cells’ infiltration, thereby reducing hepatocyte degeneration. Intriguingly, serum IF-γ level showed a significant increase to 155.92 ± 10.12 ng/L with combined therapy, reflecting the immunological role of the combined therapy. The current results revealed that probiotics have a high adjuvant potential in alleviating the impact of toxoplasmosis. Using probiotics as a synergistic treatment to modulate conventional therapy in systemic toxoplasmosis may gain popularity due to their low cost and current availability.

## Introduction

Toxoplasmosis is a global zoonotic disease caused by the protozoan *Toxoplasma gondii* (*T. gondii*) (de Lima Bessa et al. 2021). It is one of the most far-flung parasites due to its wide range of host species and extensive ability to be transmitted between hosts via multiple mechanisms (Paul et al. 2018). Toxoplasmosis represents a significant health threat to both humans and livestock, inducing high morbidity and economic losses (Rouatbi et al. 2019). It can cause life-threatening infections in immunocompromised persons and developing fetuses (Harker et al. 2015). The organism’s virulence, its genetic background, and the host’s immunological status have a significant impact on the course of infection in human and animal models of toxoplasmosis (Manuel et al. 2020). Hence, the immune responses to *T. gondii* infection can markedly affect the course and severity of infection (Maraghi et al. 2019).

Various therapies can be used for toxoplasmosis treatment. Initially, pyrimethamine and sulfadiazine were used with leucovorin. However, they had many drawbacks, including toxic effects and limited accessibility, besides their high costs. Furthermore, they were not effective against the tissue cysts (Alday and Doggett 2017). Later, sulfadiazine was replaced with clindamycin, which was less effective in preventing relapse and had similar toxicity rates (Elmehy et al. 2021). Other treatment options include atovaquone or azithromycin, which have been studied to be used as an alternate therapy. Nevertheless, they had similar rates of patient intolerance. In contrast, the trimethoprim-sulfamethoxazole (SMZ-TMP) combination demonstrated greater efficacy during toxoplasmosis treatment compared to pyrimethamine-sulfadiazine. Additionally, this combination has been proven highly effective, with fewer side effects than other combination therapies. Moreover, it can diffuse into the central nervous system (FarahatAllam et al. 2020), and fortunately, it is also available and affordable in many developing countries (de Kock et al. 2018).

Probiotics have been found to play a crucial role in the development, maturation, and modulation of the immune system during infections, so the authors of the present study considered incorporating them into the current therapeutic regimen in an effort to control parasite replication and enhance the host immune response (Yan and Polk 2011; Wang et al. 2021). Probiotics are intestinal microbiota with immunomodulatory benefits. Therefore, they have gained considerable attention and were therapeutically employed as a treatment for ailments caused by pathogenic microorganism (Azad et al. 2018).

Coupled with the prior, previous trials for treating parasitic infections using probiotics interestingly resulted in a significant reduction of the pathogenicity of multiple parasites (Berrilli et al. 2012). Probiotics recorded significant potential against protozoa such as *Giardia duodenalis*, *Cryptosporidium parvum*, and *Eimeria tenella* (Giannenas et al. 2012); nematodes such as *Toxocara canis* and *Strongyloides stercoralis* (Oliveira-Sequeira et al. 2014); and trematodes as *Schistosoma mansoni*, generating promising results (El-Khadragy et al. 2019). These promising results prompted the current study authors to evaluate the role of probiotics as adjuvant therapy in the treatment of acute experimental toxoplasmosis.

## Materials and methods

### Setting and timing of the study

The current experimental study was performed at the Medical Parasitology Department, Faculty of Medicine, Alexandria University, in cooperation with the Medical Parasitology, Biochemistry, and Pathology Departments, Faculty of Medicine, Tanta University, from June 2021 to May 2022.

### Preparation of *Toxoplasma gondii* tachyzoites

The RH virulent strain of *T. gondii* was obtained from the Medical Parasitology Department, Faculty of Medicine, Alexandria University, Egypt. In Swiss albino mice, the parasite life cycle was maintained through serial intraperitoneal (IP) passages of tachyzoites in Swiss albino mice. The tachyzoites were collected from the peritoneal fluids of the infected mice on the 7th day post-infection (p.i.). Then, tachyzoites were washed three times and diluted with saline. The mice were infected by IP injection at a dose of 2.5 × 10^3^ tachyzoites/mouse. Tachyzoite count was performed using a hemocytometer, according to Ralte et al. (2017).

### Experimental animals

This study included 90 male Swiss albino mice, free of parasites, aged 3–5 weeks, and weighing 20–25 g. Mice were maintained on a commercial pellet diet and water at a conventional room temperature of about 26 °C. The mice were divided into five groups as follows:

- Group I (negative control): 10 uninfected untreated control mice.
- Group II (positive control): 20 infected untreated control mice.
- Group III: 20 mice infected and treated with SMZ-TMP (100 mg/kg per day).
- Group IV: 20 mice infected and treated with *Lactobacillus* probiotic (1 billion per day).
- Group V: 20 mice infected and treated with a combination of SMZ-TMP (100 mg/kg per day) and *Lactobacillus* probiotics (1 billion per day).

All groups, except group I mice, were intraperitoneally infected at a dose of 2.5 × 10^3^ tachyzoites/mouse.

### Drug regimens

#### Lactobacillus probiotics

They were purchased from Adare Pharmaceuticals, Egypt, batch no. 183261 and were given orally at a dose of one billion *Lactobacilli* per mouse, administered daily for 14 days, 7 days before infection, and 7 days p.i. (Salas-Lais et al. 2020). They were prepared by dissolving each probiotic sachet that contains 10 billion *Lactobacillus delbrueckii* and *Lactobacillus fermentum* in 1 mL of distilled water to prepare the required concentration. Each mouse was given 0.1 mL of the prepared probiotics.

#### Sulfamethoxazole-trimethoprim (SMZ-TMP) (Septazole®)

This was purchased from Alexandria Co. for Pharmaceuticals and Chemical Industries and was given orally at a dose of 100 mg/kg/day. Treatment started from the first day of infection and continued daily for 7 days (Bottari et al. 2015). Each Septazole tablet of 480 mg was crushed and dissolved in 20 mL of distilled water to prepare the required concentration as needed, and each mouse was given 0.1 mL of the prepared suspension.

### Clinical study

Mice were observed daily to record any change in their food intake or clinical behavior (including attitude and posture) (Hagras et al. 2019).

### Parasitological examination

- Survival rate: It was calculated by dividing the number of survived mice at sacrifice time/number of mice at the beginning of the experiment × 100, according to Eissa et al. (2012).
- Survival time: It was calculated by daily observation of mice to determine the percentage of mice living over time (Zhao et al. 2017).
- Parasite load (mean tachyzoites count): It was estimated in liver impression smears stained with Giemsa stain. The mean number of tachyzoites was calculated in thirty different fields using an oil immersion lens (× 1000). Subsequently, the mean number of each infected group was determined according to Elgawad et al. (2019).
- Percent reduction (%R): The following equation was used to calculate the reduction in the parasite burden in the liver specimens of the experimental groups (El-Zawawy et al. 2015).
  $$\%\mathrm R=\frac{\mathrm{Mean}\;\mathrm{number}\;\mathrm{of}\;\mathrm{tachyzoites}\;\mathrm{in}\;\mathrm{control}\;\mathrm{group}-\mathrm{Mean}\;\mathrm{number}\;\mathrm{of}\;\mathrm{tachyzoites}\;\mathrm{in}\;\mathrm{infected}\;\mathrm{group}\times100}{\mathrm{Mean}\;\mathrm{number}\;\mathrm{of}\;\mathrm{tachyzoites}\;\mathrm{in}\;\mathrm{the}\;\mathrm{control}\;\mathrm{group}}$$
- Morphological study: The collected peritoneal fluid of the studied groups was examined using SEM for the detection of the morphological changes of *T. gondii* tachyzoites collected on the 7th day p.i. The peritoneal fluid was washed twice with saline. It was later fixed in glutaraldehyde. The samples were washed three times by flooding with large volumes of sterile distilled water, and then processed (Ramírez-Flores et al. 2019; Gamea et al. 2022) and examined using a JEOL JSM-IT200 scanning microscope in the Alexandria electron microscopy unit Alexandria Faculty of Science.

### Histopathological examination

Liver specimens from mice were collected for histopathological examination. They were fixed in 10% formalin before being embedded in paraffin and then, serial sections (5 µm thick) were cut using a microtome. They were stained by hematoxylin and eosin (H&E), as described by Carleton et al. (1980), and were examined by the light microscope for assessment.

### Immunological study

The interferon-gamma (IFN-γ) level was determined in the sera of all studied groups using a commercially available ELISA kit (Sunred Biotechnology Pharmaceuticals Company, China) in accordance with the manufacturer’s instructions.

### Statistical data analysis

The collected data were analyzed using SPSS software (Statistical Package for Social Sciences) version 20. All values were expressed as mean ± standard deviation. The significance of differences between the groups was calculated using Student’s *F* test. The level of statistical significance was set at a *P* value of < 0.05.

## Results

### Clinical and behavior studies

Compared to the uninfected mice, the infected untreated mice group showed diminished food intake and lethargic behavior starting from the 5th day p.i., with ruffled fur and hunched posture. Contrarily, the infected treated mice groups appeared relatively healthy with relatively normal food intake. The activity of mice in the SMZ-TMP and *Lactobacillus* probiotics–treated group (group V) showed more significant improvement compared to other treated groups.

### Parasitological examination results

#### Survival rate (SR) and survival time (ST)

**Fig. 1:**
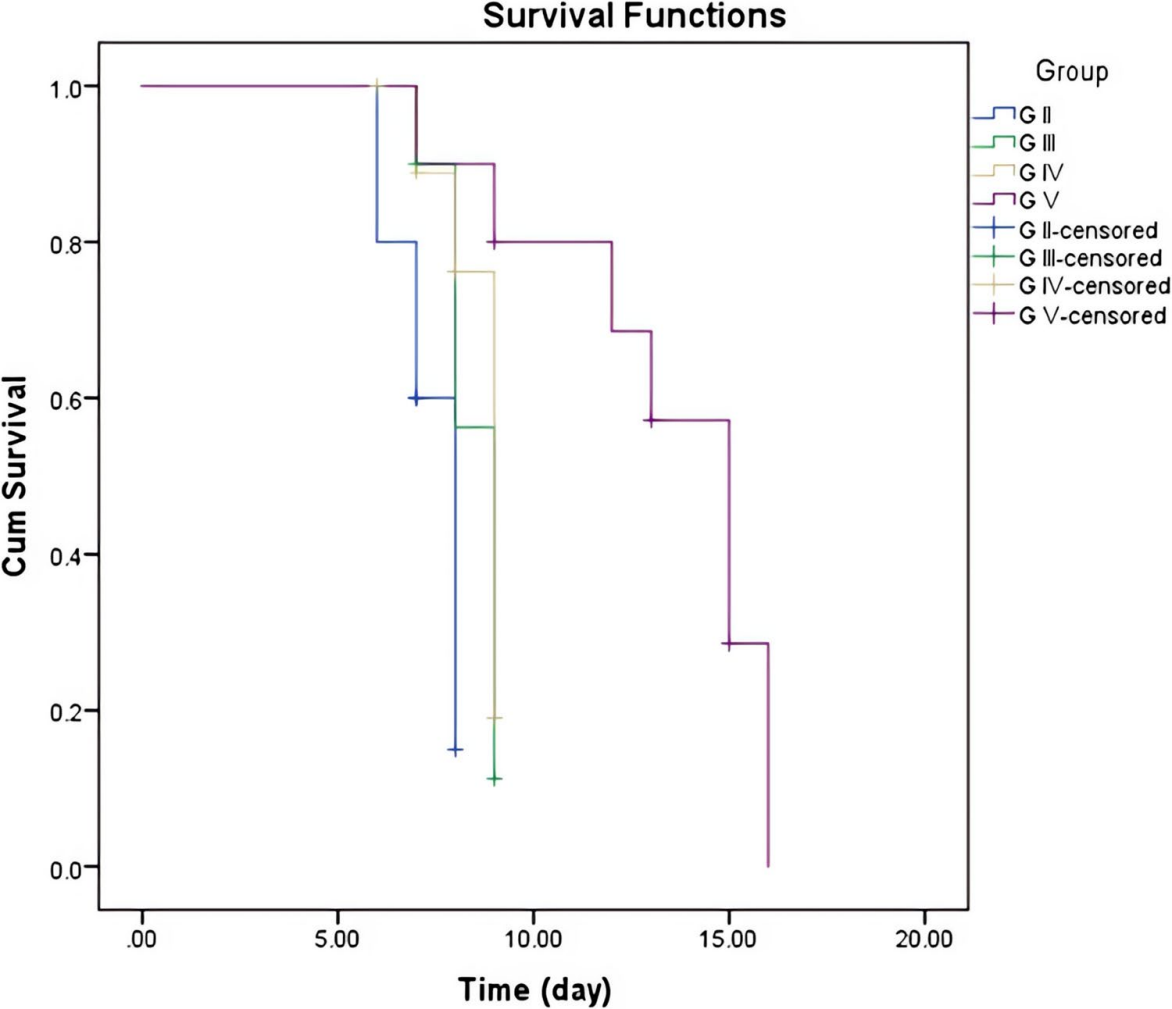
Kaplan–Meier overall survival curve of the studied groups

In comparison to the infected untreated control group, the SR has increased in all treated groups. The survival rate was 70% in the infected untreated control group (II). Conversely, survival rates were 90%, 85%, and 95% in groups III, IV, and V, respectively, with no significant difference (*P* = 0.139) (Table 1). Regarding the ST, the maximum ST was 8 days in group II and 9, 9, and 16 days in groups III, IV, and V, respectively, with the highest, reported ST in group V, which received the combined therapy (Fig. 1).

**Table 1:** The survival rate in the studied groups

	G-II		G-III		G-IV		G-V	
	Number	Percent	Number	Percent	Number	Percent	Number	Percent
Survived	14	70%	18	90%	17	85%	19	95%
Died	6	30%	2	10%	3	15%	1	5%
Total	20	100%	20	100%	20	100%	20	100%
***χ***^2^	5.489							
*P* value	0.139							

*χ*^2^, chi-square test

*G-II*, control non-treated; *G-III*, SMZ-TMP; *G-IV*, Lb; *G-V*, SMZ-TMP + Lb

#### Parasite load and percent reduction (%R)

**Fig. 2:**
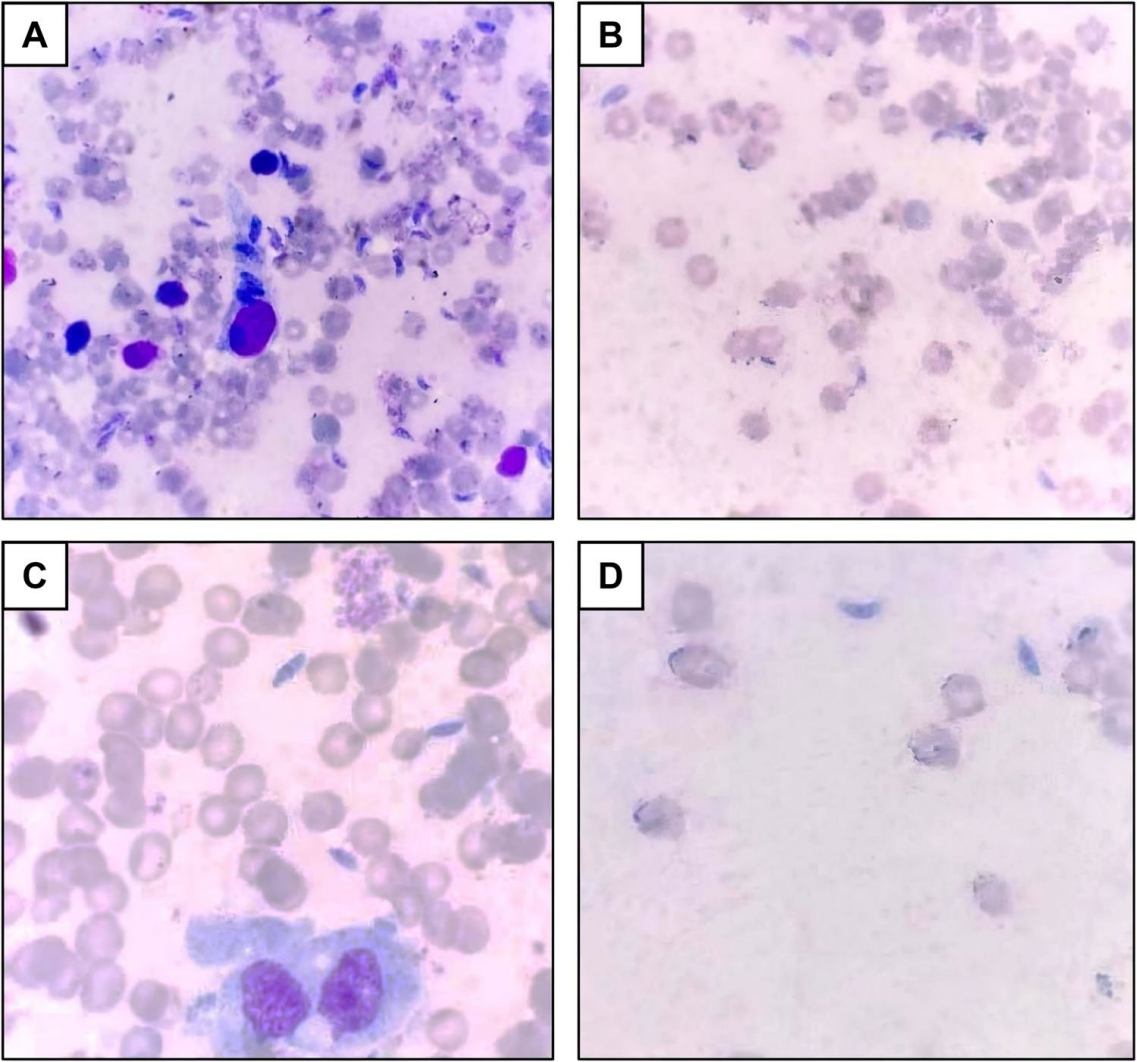
Giemsa-stained tachyzoites in the impression smears (× 1000) obtained from mice livers of groups II, III, IV, and V (**A**, **B**, **C**, **D**), respectively, showing the highest percent reduction in the number of tachyzoites in group V

There was a statistically significant reduction (*P* = 0.001) in the mean number of the tachyzoites in the impression smears obtained from mice livers of the treated groups III, IV, and V (6.75, 8.25, and 2.22, respectively) compared to the infected untreated control group II (12.38) (Table 2; Fig. 2). The highest percent reduction was obtained in the group treated with the combined therapy. There was a statistically significant reduction difference between group V and other treated groups (*P* = 0.001) and between groups III and IV (*P* = 0.002).

**Table 2:** The mean parasitic count in the mice’ livers of the studied groups

	G-II	G-III		G-IV		G-V
Range	10.5–14.1	5.2–8.5		6.8–9.4		1.2–3.1
Mean ± SD	12.38 ± 1.27	6.75 ± 1.07		8.25 ± 0.89		2.22 ± 0.54
%R		45.4%		33.3%		82%
*F* test	183.562					
*P* value	0.001*					
P1	P2	P3	P4		P5	P6
0.001*	0.001*	0.001*	0.002*		0.001*	0.001*

*P1*, *P* value comparison between groups II and III; *P2*, *P* value comparison between groups II and IV; *P3*, *P* value comparison between groups II and V; *P4*, *P* value comparison between groups III and IV; *P5*, *P* value comparison between groups III and V; *P6*, *P* value comparison between groups IV and V; *SD*, standard deviation; *%R*, percent reduction; *F* test, ANOVA test

^*^Significant (*P* < 0.05)

*G-II*, control non-treated; *G-III*, SMZ-TMP; *G-IV*, Lb; *G-V*, SMZ-TMP + Lb

#### Morphological study results

**Fig. 3:**
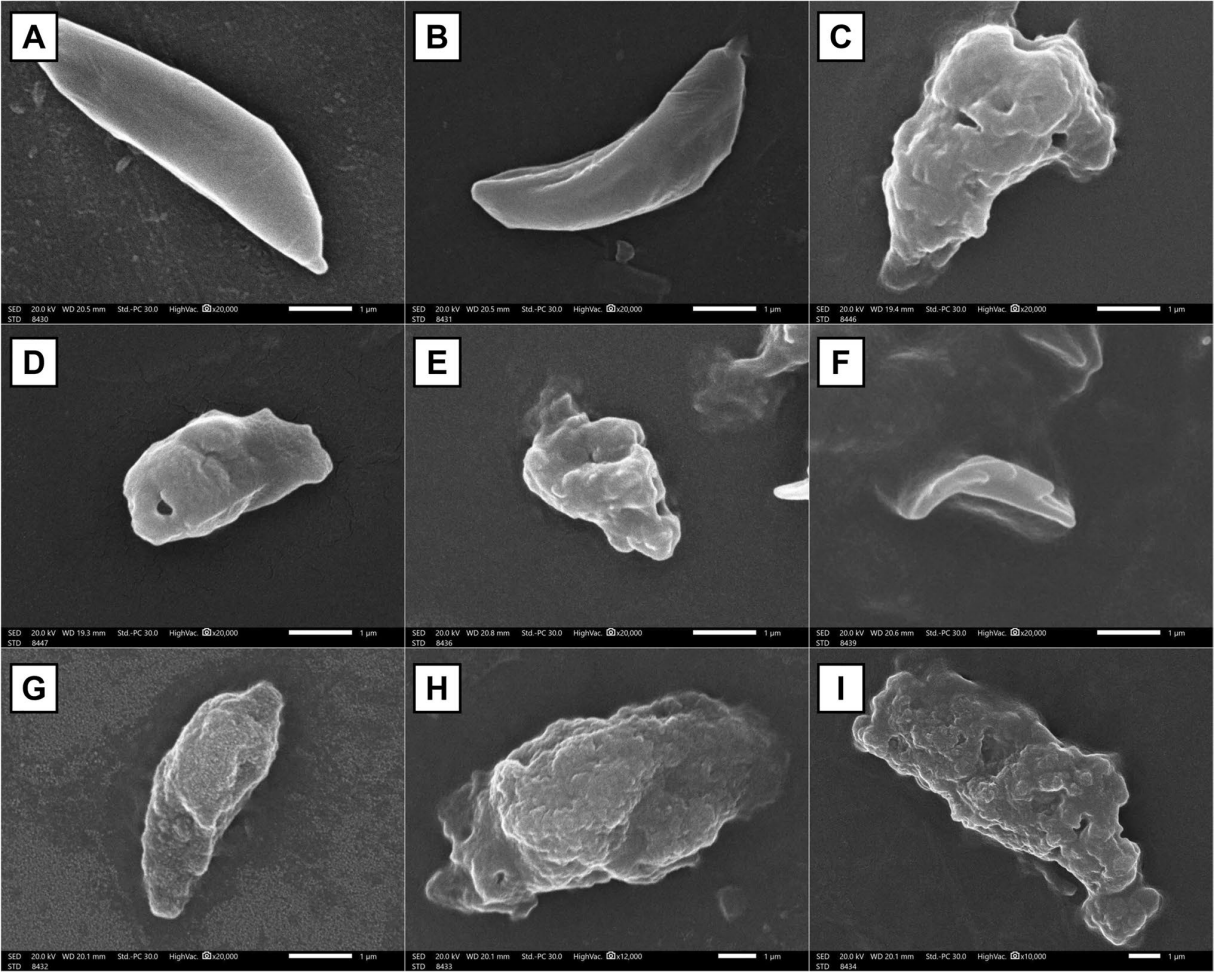
SEM of *T. gondii* tachyzoites in the studied groups. Group II (**A**, **B**) (× 20,000): tachyzoite showing a typical usual crescent shape of tachyzoites. Group III (**C**, **D**) (× 20,000): tachyzoite showing distorted crescent shape, protrusions, and holes on the surface with the presence of multiple blebs at the tachyzoite surface and a hole at one of the tachyzoite end. Group IV (**E**, **F**) (× 20,000): tachyzoite showing distorted crescent shape, multiple irregularities on the surface, and reduction in size. Group V (**G** (× 20,000), **H** (× 12,000), **I** (× 10,000)): tachyzoite showing distorted crescent shape, erosions, and elevations on the tachyzoite surface with the presence of multiple holes on the surface, ballooning, and increase of the tachyzoite size

Scanning electron microscopic imaging of *T. gondii* tachyzoites obtained from the peritoneal fluid of the untreated group revealed elongated, crescent-shaped, smooth-surfaced tachyzoites, with a rounded pole at one end and a pointed pole at the other one (evident conoid) (Fig. 3A, B).

In contrast, the tachyzoites from the SMZ-TMP-treated group showed distorted crescent-shaped tachyzoites, with loss of their smooth surface. Additionally, surface protrusions, holes, and their ends were distorted (Fig. 3C). Multiple surface blebs were also observed (Fig. 3D). Tachyzoites from the *Lactobacillus*-treated group showed distorted crescent shape, multiple surface irregularities, and reduced size. The distortion of their ends appeared as ruptured tachyzoites (Fig. 3E, F). The tachyzoites from the combined therapy–treated group werealso distorted, with surface erosions and elevations (Fig. 3G). Multiple holes, ballooning, and increased size were also noted (Fig. 3H, I).

#### Histopathological study results

**Fig. 4:**
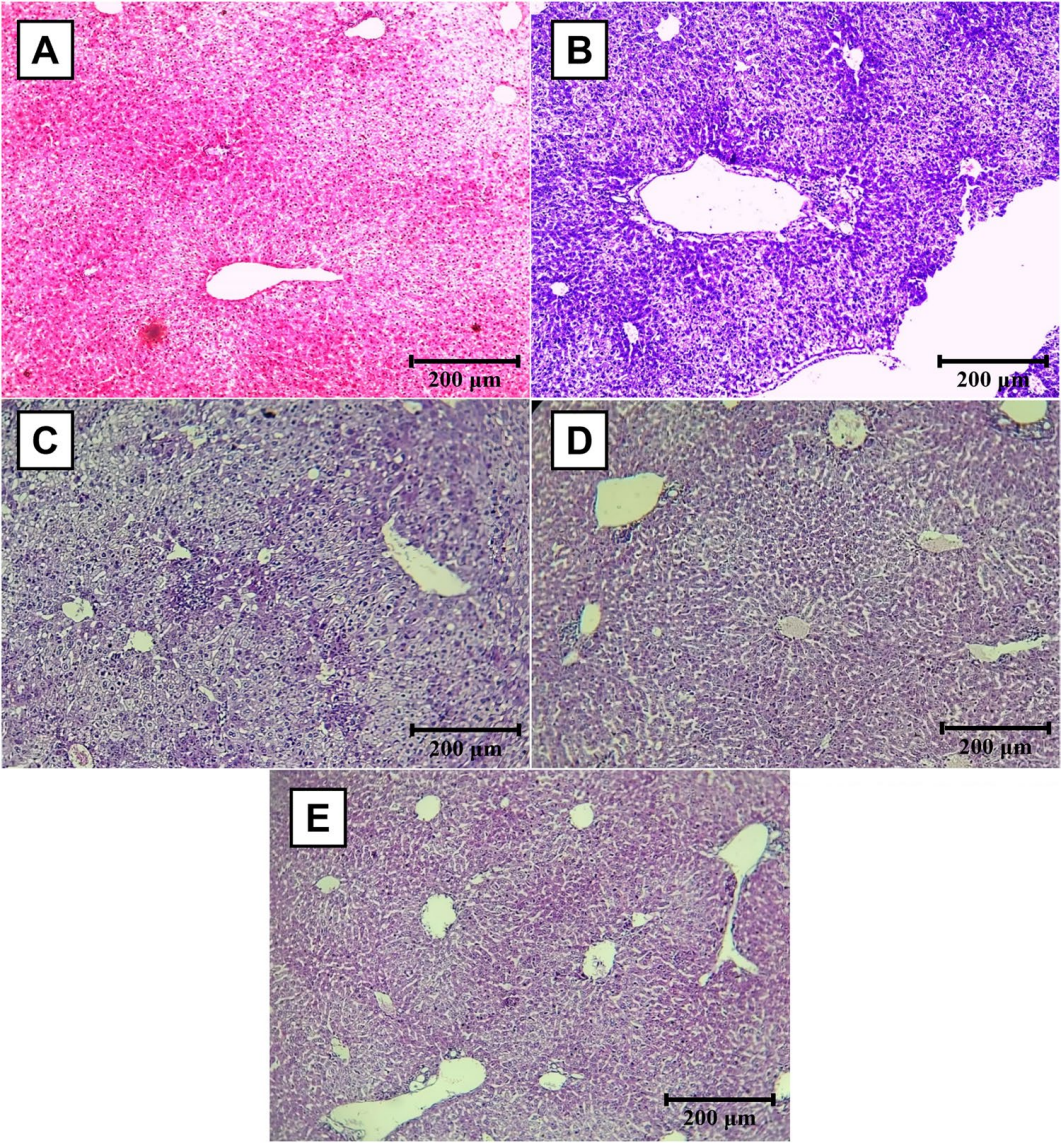
Hematoxylin and eosin–stained liver sections of the studied groups (H&E × 100). **A** Uninfected untreated mice showing normal histological features of the liver. **B** Infected untreated mice showing marked inflammatory changes in the liver characterized by heavy infiltration of portal tracts by inflammatory cells and hydropic degeneration of the hepatocytes. The central veins were congested and surrounded by inflammation. **C** SMZ-TMP-treated mice showing relatively reduced inflammatory changes in the liver tissue showing moderate infiltration of portal tracts by inflammatory cells and congestion of the central veins. **D** *Lactobacillus*-treated mice showing a relative increase in the infiltration of portal tracts by inflammatory cells but with decreased hydropic degeneration of the hepatocytes. **E** Combined treated mice showing improved histopathological features with mild infiltration of portal tracts by lymphocytes

In the healthy control group, mice liver sections revealed normal polygonal hepatocytes with rounded central nuclei. Hepatocytes were organized in two-cell-thick trabeculae radiating from the central veins. The portal tracts appeared normal (Fig. 4A). Conversely, the infected untreated control group showed extensive lymphocytic inflammatory cell infiltration surrounding the portal tracts, as well as congestion of the central veins and ballooning of the hepatocytes due to hydropic degeneration (Fig. 4B).

Comparable to the infected untreated group, the *Lactobacillus*-treated group demonstrated an increase in inflammatory infiltrates within the portal tracts, but with decreased hydropic degeneration of the hepatocytes and decreased portal tract congestion (Fig. 4C). In addition, other treated groups demonstrated a decrease in the severity and extent of inflammatory cellular infiltrates, as well as a decrease in hepatocyte degeneration, compared to the infected untreated control group. This decrease was most pronounced in group V (Fig. 4D, E) compared to the SMZ-TMP-treated group.

#### Immunological study results

The results revealed that the uninfected control group exhibited the lowest noticeable levels of serum IFN-γ, followed by the SMZ-TMP-treated group. In contrast, mice of the *Lactobacillus*-treated group exhibited the highest level of IFN-γ production, followed by the group treated with the combined therapy. The serum IFN-γ levels’ differences were statistically significant (*P* = 0.001) in the studied groups. There was a statistically significant difference in the untreated and treated groups in comparison to the uninfected control group. Moreover, a statistically significant difference was detected between the infected untreated group and the three treated groups (Table 3).

**Table 3:** The serum IFN-γ levels in the experimental groups

Serum IFN-γ statistics (ng/L)		G-I			G-II			G-III		G-IV			G-V	
Range		6.9–10.1			108.7–157.7			100.5–134.2		154.1–183.6			142.5–167.1	
Mean ± SD		8.35 ± 1.14			136.00 ± 16.35			120.90 ± 12.98		171.37 ± 10.35			155.92 ± 10.12	
*F* test		193.052												
*P* value		0.001*												
P1	P2		P3	P4		P5	P6		P7		P8	P9		P10
0.001*	0.001*		0.001*	0.001*		0.030*	0.001*		0.006*		0.001*	0.001*		0.027*

*P1*, *P* value comparison between groups I and II; *P2*, *P* value comparison between groups I and III; *P3*, *P* value comparison between groups I and IV; *P4*, *P* value comparison between groups I and V; *P5*, *P* value comparison between groups II and III; *P6*, *P* value comparison between groups II and IV; *P7*, *P* value comparison between groups II and V; *P8*, *P* value comparison between groups III and IV; *P9*, *P* value comparison between groups III and V, *P10*, *P* value comparison between groups IV and V

^*^Significant (*P* < 0.05)

*G-I*, control normal; *G-II*, control non-treated; *G-III*, SMZ-TMP; *G-IV*, Lb; G-V, SMZ-TMP + Lb

## Discussion

*Toxoplasma gondii* is thought to be one of the globally significant apicomplexan protozoa that are highly impacting people all over the world (Nazari et al. 2018). Notably, the severity of toxoplasmosis is mainly affected by the host immune system integrity (Mamaghani et al. 2022).

Due to the immunological impact of probiotics, we believe they can significantly contribute to improving the current therapeutic regimens of toxoplasmosis by enhancing the host’s immune response to infection. Several works have studied the role of probiotics in combatting infections. The potential immunomodulatory effect of *Lactobacillus casei* as a probiotic in experimental trichinosis was reported by El Nouby et al. (2004). Gaber et al. (2022) reported the effectiveness of commercial *Lactobacillus* probiotics on experimental mice challenged with *Cryptosporidium parvum* and reported their immunomodulatory role. Moreover, Ribeiro et al. (2016) observed that probiotic treatment has a promising immunomodulatory activity against the *T. gondii* ME49 strain. In the same context, the present study showed the potential immunomodulatory, and therapeutic effects of *Lactobacillus* probiotics on acute experimental toxoplasmosis in mice were evaluated and compared to the SMZ-TMP combination.

In clinical and behavioral studies of mice, the combination of SMZ-TMP and probiotics resulted in a significant reduction in signs of pain and distress in rodents compared to the infected untreated mice. These improvements in the clinical signs in the current study matched the same observations documented by Teimouri et al. (2018), Gomaa et al. (2019), and El-Sayad et al. (2020), who studied RH *T. gondii* infection in murine models.

With regard to the SR and ST, the current research found that, in the infected untreated group, no mice survived after the 8th day p.i. This finding was supported by El Temsahy et al.’s (2016) and FarahatAllam et al.’s (2020) research results on using the same dose of infection (2.5 × 10^3^ tachyzoites). On the contrary, the highest SR and the longest ST were reported in SMZ-TMP, and probiotic combined–treated group (95% and 16 days, respectively). Consistent with the current results, Elmehy et al. (2021) reported enhanced SR of mice treated with immunomodulatory adjuvant therapy. Contrarily, there was a difference in ST and SR on comparing these results with other experimental studies. These differences can be attributed to the variance in infection dose and other environmental factors (Teimouri et al. 2018).

Concerning the parasite load, there was a significant reduction in the mean tachyzoite count in the liver impression smears in the treated mice groups compared to the untreated group. The reduction percentage of parasite burden in the SMZ-TMP-treated group was 45.4%, which is consistent with FarahatAllam et al.’s (2020) study results, taking into consideration the same infective dose and the same SMZ-TMP treating dose. Interestingly, *Lactobacillus* probiotics showed an anti-*Toxoplasma* effect, as the parasite load was 33% in the *Lactobacillus* probiotics–treated group. The highest reduction of parasite burden was detected in the combined treated group (82%), which might explain the synergistic action of *Lactobacillus* probiotics. Salas-Lais et al. (2020) also reported a reduction of tachyzoite count in peritoneal exudate after 14 days of treatment by *Lactobacillus casei* in systemic murine toxoplasmosis.

In addition, SEM of tachyzoites from all treated mice groups showed remarkable morphological changes that were more evident in the combined treated group. Similar ultrastructural changes were reported by Sanfelice et al.’s (2021) study upon using biogenic silver nanoparticles in toxoplasmosis treatment. FarahatAllam et al. (2020) suggested that these ultrastructural changes affect the reproduction of the tachyzoites, leading to a significant decrease of the parasite load in liver impression smears of treated groups, with a consequent increase in SR and ST. Hammouda et al. (1992) hypothesized that the morphological changes in the organisms could be due to changes caused by compounds interfering with the parasite DNA synthesis or the folic acid cycle. Gaafar et al. (2022) likewise documented that the deformities of the tachyzoites with disorganization of the conoids and the significant reduction in the parasite load in the impression smears were described as the anti-*Toxoplasma* effect.

Within the context of the histopathological study, the present study revealed that the treatment with *Lactobacillus* probiotics was associated with a relative increase in the infiltration of hepatic portal tracts by inflammatory cells compared with other treated groups. This finding aligns with Vitiñi et al. (2000), who stated that most *Lactobacillus* probiotics have no cytotoxic activity but can induce inflammatory immune responses. This observation was subsequently reconsidered by Livingston et al. (2010), who observed a mild transitory inflammatory response associated with *Lactobacillus* probiotics in the murine model within 6 days, which subsided within 21 days.

In order to validate the obtained results, serum IFN-γ was measured in all experimental groups. The highest level of serum IFN-γ was found in the *Lactobacillus* probiotics–treated group. This result emphasizes the immunomodulatory role of *Lactobacillus* probiotics and strongly clarifies the results of reduction in the parasite load in this group. Similarly, Azad et al. (2018) reported the probiotics’ ability to influence host immunity by IFN-γ cytokine induction. Furthermore, Salas-Lais et al. (2020) found that probiotics are potent enhancers to increase cytokine levels in the infected mice ascites fluid. The increase of serum IFN-γ level in the infected untreated group can be significantly attributed to the immune response to infection (Azad et al. 2018). This level decreased with SMZ-TMP treatment, which can be attributed to its anti-*Toxoplasma* activity, which is compatible with the findings of Soheilian et al. (2005) and Francis et al. (2004) on both ocular and cerebellar toxoplasmosis, respectively.

Focusing on the role of IFN-γ, the major cytokine of T-helper-1 plays a crucial role in protecting against intracellular parasites (Sibley 2011). It enhances phagocytosis by stimulating macrophages to eliminate intracellular pathogens through the production of inflammatory mediators such as reactive oxygen. According to Sanfelice et al. (2021), protection against or susceptibility to *T. gondii* infection is primarily determined by the cytokines that modulate and direct the immune response. Moreover, according to Cristofori et al. (2021), the interaction between probiotics, gut epithelial cells, and immune cells leads to an increase in specific cytokines’ profiles, which aims to regulate the immune response.

The current experimental study aimed to establish a rationale for the use of *Lactobacillus* probiotics, which are safe, inexpensive, and commercially available compounds that can be used as an adjuvant therapy to improve the prognosis of toxoplasmosis. This concept is novel and may become simple and widespread. The results of this study indicate that *Lactobacillus* probiotics can potentially mitigate the effects of *T. gondii* infection on experimental mice. The administration of combined therapy may result in a significant reduction in the dosages and side effects of conventionally administered drugs. Therefore, additional research is still required, and a formula containing a combination therapy would be essential to be provided in clinical practice.

## Conclusion

*Lactobacillus* probiotics showed a promising immunomodulatory effect against acute toxoplasmosis; this was shown when used alone and in combination with SMZ-TMP, though it was more effective when combined with SMZ-TMP. This result was observed through the relatively normal appearance of mice, the reduction in parasite burden in the liver, the ultrastructural changes of trophozoites by SEM, the improvement of histopathological changes in the liver, and the modulation of serum IFN-γ level.

## Data Availability

Not applicable.
